# Partitioning the forms of genotype-by-environment interaction in the reaction norm analysis of stability

**DOI:** 10.1007/s00122-023-04319-9

**Published:** 2023-04-07

**Authors:** Dominic L. Waters, Julius H. J. van der Werf, Hannah Robinson, Lee T. Hickey, Sam A. Clark

**Affiliations:** 1 School of Environmental and Rural Science, University of New England, Armidale, NSW 2351 Australia; 2 InterGrain Pty Ltd, Perth, WA Australia; 3 Queensland Alliance for Agriculture and Food Innovation, The University of Queensland, Brisbane, QLD Australia

## Abstract

**Key message:**

The reaction norm analysis of stability can be enhanced by partitioning the contribution of different types of G × E to the variation in slope.

**Abstract:**

The slope of regression in a reaction norm model, where the performance of a genotype is regressed over an environmental covariable, is often used as a measure of stability of genotype performance. This method could be developed further by partitioning variation in the slope of regression into the two sources of genotype-by-environment interaction (G × E) which cause it: scale-type G × E (heterogeneity of variance) and rank-type G × E (heterogeneity of correlation). Because the two types of G × E have very different properties, separating their effect would enable a clearer understanding of stability. The aim of this paper was to demonstrate two methods which seek to achieve this in reaction norm models. Reaction norm models were fit to yield data from a multi-environment trial in Barley (*Hordeum vulgare*), with the adjusted mean yield from each environment used as the environmental covariable. Stability estimated from factor-analytic models, which can disentangle the two types of G × E and estimate stability based on rank-type G × E, was used for comparison. Adjusting the reaction norm slope to account for scale-type G × E using a genetic regression more than tripled the correlation with factor-analytic estimates of stability (0.24–0.26 to 0.80–0.85), indicating that it removed variation in the reaction norm slope that originated from scale-type G × E. A standardisation procedure had a more modest increase (055–0.59) but could be useful when curvilinear reaction norms are required. Analyses which use reaction norms to explore the stability of genotypes could gain additional insight into the mechanisms of stability by applying the methods outlined in this study.

**Supplementary Information:**

The online version contains supplementary material available at 10.1007/s00122-023-04319-9.

## Introduction

Genotype-by-environment interactions (G × E) occur when the performance of a genotype is dependent on the environment it exists in. The performance of some genotypes can be more sensitive to environmental factors, whilst others show greater stability across a range of environments. Genotypes that combine high overall performance with stability are appealing for their greater marketing potential and utility in mitigating the effects of climate change on crop production (Powell et al. 2012). Therefore, there is a need to understand the models available to estimate genetic variation and select parent genotypes for stability in plant breeding programs.

Reaction norm (RN) models have been a popular choice for modelling G × E and the stability of genotypes across environments (Kraakman et al. 2004; Kusmec et al. 2017; Lacaze et al. 2009; Li et al. 2019; Ly et al. 2018; Sjoberg et al. 2020). In RN models, the performance of a genotype is regressed across an environmental covariable (EC), which describes the quality of the environment as a continuous value (Woltereck 1913). If the EC is centred with a mean of zero, the intercept represents performance in the average EC, whilst the slope of the regression captures the stability of the genotype. The regression can be either fixed or random; random regression is more common, as additive relationships can then be accounted for via pedigree or genomic data, which enables the estimation of additive genetic breeding values and genomic predictions of stability. The current paper focuses on modelling additive genetic random regression RN models, although the concepts are relevant to all types of RN models.

There are two types of G × E which can contribute to variation in the slope of a RN model, and both have very different properties. Rank-type G × E occurs when the genetic correlation between environments is less than unity. A correlation significantly less than unity means that between environments, genotypes re-rank and the relative effect of the underlying genes changes (Falconer 1952). In contrast, scale-type G × E occurs when the genetic variance changes between environments. This “scales” the breeding values such that the differences between genotypes get larger or smaller, but it does not affect the genetic correlation (James 2009). Separating the two sources of variation in the slope of a reaction norm would enable a clearer picture of re-ranking between genotypes, and how the genetic architecture of a trait varies across the EC. If the effect of scale-type G × E was removed from the reaction norm slope, the resulting slope would capture how much the genotype re-ranks across the EC, or its “rank-stability”. This could assist breeders to select genotypes which combine high overall performance with a stable ranking across a given EC, or alternatively, genotypes with adaptation to a specific EC level.

A method suggested by Falconer (1990) proposes that for a linear RN model with the intercept placed at the average EC, the square of the correlation between the intercept and slope (*r*^*2*^) represents the proportion of variance in the slope that is due to scale-type G × E. As such, the proportion of variance in slope due to rank-type G × E can be estimated as (1–*r*^*2*^). A genetic regression can also be used to derive breeding values for the reaction norm slope that is independent of the intercept variance (thus independent of scale-type G × E), as recently applied in the reaction norm analysis of body weight in sheep (Waters, Clark, et al. 2022).

A drawback of Falconer’s method is that it is limited to a linear function, which might not adequately describe the response of a genotype across the EC in some situations. This could be addressed by directly standardising the breeding value of genotypes across the EC to a constant genetic variance, thereby removing variation in stability due to scale-type G × E. For a given genotype, the change in standardised breeding value could then be used as an estimate of stability due to rank-type G × E.

Factor-analytic (FA) models can also separate scale-type and rank-type G × E when analysing the stability of genotypes across environments (Smith and Cullis 2018). Whilst RN models employ a single user-defined environmental covariable, FA models generate an unsupervised set of *c* common factors that represent unobserved environmental covariables which explain changes in genetic effects across environments (Meyer 2009). The slopes of regression on the common factors can be used to derive breeding values for stability whilst accounting for G × E (Smith and Cullis 2018). A downside of using FA models is that they reveal unobserved environmental factors which may not always be interpretable. Therefore, the model is less useful than RN models when investigating a specific environmental factor, such as in designed experiments. However, the ability of FA models to separate scale-type and rank-type G × E could be leveraged to investigate scale-correction methods for RN models.

The aim of this study was to apply the two methods for separating variation due to scale and rank-type G × E when estimating breeding values for stability from random regression RN models. The dataset involves a MET from an Australian barley breeding program with large amounts of scale-type and rank-type G × E. The resulting breeding values for stability from the RN models were compared with those derived from FA models to test how well they accounted for scale-type G × E. We use genomic data to model the additive genetic relationships amongst genotypes.

## Methods

### Phenotypic data

The data were provided by InterGrain Pty Ltd (www.intergrain.com) and consisted of plot yields from 52 partially replicated trials distributed across 15 locations throughout Western Australia, New South Wales, Victoria, and South Australia. The trials took place in 2019 (10 trials) and 2020 (42 trials).

There were 3460 unique genotypes with yield phenotypes, although 2734 of the genotypes were only represented in one environment and did not have single nucleotide polymorphism (SNP) data. The remaining 726 genotypes were almost fully replicated across the 15 environments and had SNP data.

### SNP data

The SNP data were collected using the Illumina Infinium Wheat Barley 40 K SNP array Version 1.0 and was imputed to a set of 410 499 SNPs, as described in Keeble-Gagnère et al. (2021). After imputation, SNPs with a minor allele frequency less than 0.05 (103 489 SNPs) and with more than 12.5% heterozygosity (202 SNPs) were removed, leaving a total of 306 808 SNPs for the 726 genotypes.

### Analysis

Because there was a large number of genotypes without replication across environments, a two-stage analysis was used (Smith et al. 2001) rather than a usually preferable single stage. This allowed all the genotypes to be used for modelling spatial variation within the trials in the first stage, whilst avoiding the inclusion of 2734 uninformative genotypes to model G × E across environments in the second stage. All the models were fit using restricted maximum likelihood (REML) in ASReml-R (Butler et al. 2018).

In the first stage, trials were analysed independently to obtain best linear unbiased estimates (BLUEs) of yield for each genotype, along with the corresponding weights. For each trial, the initial model consisted of an independent random genotype effect and a first-order separable autoregressive term for the error variance structure using the row and column dimensions of the trial. Based on this model, plots with mean yields deviating more than four standard deviations from their expectation were removed. Additional random ‘row’ and ‘column’ terms were fitted based on a log-likelihood ratio test with a threshold of *p* = 0.05. Once the final model was determined, the BLUEs and their corresponding weights were obtained by fitting genotype as a fixed effect, following the methodology of Smith et al., (2001).

In the second stage, the performance of genotypes across the environments was analysed with RN and FA models utilising the BLUEs of yield and corresponding weights obtained in the first-stage analysis. Only the 726 genotypes with replication across environments and SNP data were considered in this stage. Of the 726 genotypes, 74 were removed from the analysis, as they were heterozygous for more than 12.5% of their SNPs. This left 652 genotypes, of which 606 were replicated across all 15 environments, with an additional 45 genotypes replicated in 14 environments. The remaining genotype was replicated in only six environments but was included in the analysis since it would contribute information through its genomic relationship to the population.

### Reaction norm models

The RN models require an environmental covariable (EC) that captures the environmental quality. Whilst it is increasingly common for ECs to consist of real environmental covariables, this was not available in our dataset. Instead, a joint-regression analysis was used (Eberhart and Russell 1966; Finlay and Wilkinson 1963; Perkins and Jinks 1968), where the adjusted mean yield of each environment was used as the EC. RN models based on JR analysis have been used widely to investigate the genetic basis of stability for yield and quality traits in cereal crops (Calderini and Slafer 1999; Kraakman et al. 2004; Li et al. 2019; Sandhu et al. 2021; Tan et al. 2020). Because the distribution of genotypes across environments was slightly unbalanced in our dataset, the direct mean of yield in each environment would be a slightly biased estimator of the EC. To account for the unequal use of genotypes across environments, a simple model which fit genotypes as an independent random effect and environment as a fixed effect was used to obtain a BLUE of yield for each environment, which formed the EC. The EC values ranged from 1.45 to 4.84 tonnes per hectare (t/ha) and were standardised to values with a variance of one and a mean of zero for the RN analysis. The distribution of genotypes along the EC is given in Fig. S1.

The first RN model was a standard linear reaction norm (RN-L), which modelled the genetic effect of each genotype as a linear function of the EC. The second RN model used cubic polynomials (i.e. a nonlinear function) to model the genetic effect across the EC and was denoted RN-NL.

The linear RN-L in simple matrix form was:

1 $${\mathbf{y}} = {\mathbf{Xb}} + {\mathbf{Z}}_{1} {\mathbf{a}}_{0} + {\mathbf{Z}}_{2} {\mathbf{a}}_{1} + {\mathbf{e}}$$

where ${\mathbf{y}}$ was a vector of BLUEs for each genotype, $\mathbf{X}$ was an incidence matrix linking records to the fixed effects $\mathbf{b}$, and ${\mathbf{Z}}_{1}$ and ${\mathbf{Z}}_{2}$ were design matrices linking records to the genomic estimated breeding values (GEBVs) for the intercept (${\mathbf{a}}_{0}$) and slope (${\mathbf{a}}_{1}$), with ${\mathbf{Z}}_{1}$ containing 1’s on the diagonal and ${\mathbf{Z}}_{2}$ containing the EC value corresponding to each BLUE in $\mathbf{y}$ on the diagonal, and $\mathbf{e}$ was the residual variance.

The environment was fitted as the only fixed effect to account for differences in the mean yield between environments, and residual variance was estimated independently for each environment. The genetic variance of ${\mathbf{a}}_{0}$ and ${\mathbf{a}}_{1}$ was modelled according to:

$$\left[ {\begin{array}{*{20}c} {{\mathbf{a}}_{0} } \\ {{\mathbf{a}}_{1} } \\ \end{array} } \right]\sim {\text{N}}\left( {0,{\mathbf{G}} \otimes {\mathbf{K}}} \right)$$, where $${\mathbf{K}} = \left[ {\begin{array}{*{20}c} {\sigma_{{{\text{a}}_{0} { }}}^{2} \quad{ }\sigma_{{{\text{a}}_{1} {\text{a}}_{0} }} } \\ {{ }\sigma_{{{\text{a}}_{0} {\text{a}}_{1} }}\quad \sigma_{{{\text{a}}_{1} { }}}^{2} } \\ \end{array} } \right]$$ and **G** was a relationship matrix based on SNP data, constructed using the first van Raden method (VanRaden 2008) and $\otimes$ refers to a Kronecker product of matrices.

For RN-NL, a cubic polynomial was fitted to describe the genetic effects across the EC. The model was a simple extension of RN-L:

2 $${\mathbf{y}} = {\mathbf{Xb}} + {\mathbf{Z}}_{1} {\mathbf{a}}_{0} + {\mathbf{Z}}_{2} {\mathbf{a}}_{1} + {\mathbf{Z}}_{3} {\mathbf{a}}_{2} + {\mathbf{Z}}_{4} {\mathbf{a}}_{3} + {\mathbf{e}}$$

where ${\mathbf{Z}}_{3}$ and ${\mathbf{Z}}_{4}$ were design matrices linking records to the GEBVs for quadratic (${{\varvec{a}}}_{2}$) and cubic (${{\varvec{a}}}_{3}$) components, with the diagonal of ${\mathbf{Z}}_{3}$ and ${\mathbf{Z}}_{4}$ containing the square and cube of the EC value corresponding to the BLUE in ${\varvec{y}}$, respectively. The remaining components${\varvec{y}}$, ${\varvec{X}}$, ${\varvec{b}}$, ${{\varvec{Z}}}_{1}$, ${{\varvec{Z}}}_{2}$, ${{\varvec{a}}}_{0}$, ${{\varvec{a}}}_{1}$ and ${\varvec{e}}$ were the same as in (1). The genetic variance of ${{\varvec{a}}}_{0}$,${{\varvec{a}}}_{1}$, ${{\varvec{a}}}_{2}$ and ${{\varvec{a}}}_{3}$ were also modelled in the same way as in RN-L, where:

$$\left[ {\begin{array}{*{20}c} {{\mathbf{a}}_{0} } \\ {{\mathbf{a}}_{1} } \\ {{\mathbf{a}}_{2} } \\ {{\mathbf{a}}_{3} } \\ \end{array} } \right]\sim {\text{N}}\left( {0,{\mathbf{G}} \otimes {\mathbf{K}}} \right),{ }$$ where $${\mathbf{K}} = { }\left[ {\begin{array}{*{20}c} {\sigma_{{{\text{a}}_{0} { }}}^{2} } &\quad {{ }\sigma_{{{\text{a}}_{1} {\text{a}}_{0} }} } &\quad {{ }\sigma_{{{\text{a}}_{2} {\text{a}}_{0} }} } &\quad {{ }\sigma_{{{\text{a}}_{3} {\text{a}}_{0} }} } \\ {{ }\sigma_{{{\text{a}}_{0} {\text{a}}_{1} }} } &\quad {\sigma_{{{\text{a}}_{1} { }}}^{2} } &\quad {{ }\sigma_{{{\text{a}}_{2} {\text{a}}_{1} }} } &\quad {{ }\sigma_{{{\text{a}}_{3} {\text{a}}_{1} }} } \\ {{ }\sigma_{{{\text{a}}_{0} {\text{a}}_{2} }} } &\quad {{ }\sigma_{{{\text{a}}_{1} {\text{a}}_{2} }} } &\quad {\sigma_{{{\text{a}}_{2} { }}}^{2} } &\quad {{ }\sigma_{{{\text{a}}_{3} {\text{a}}_{2} }} } \\ {{ }\sigma_{{{\text{a}}_{0} {\text{a}}_{3} }} } &\quad {{ }\sigma_{{{\text{a}}_{1} {\text{a}}_{3} }} } &\quad {{ }\sigma_{{{\text{a}}_{2} {\text{a}}_{3} }} } &\quad {\sigma_{{{\text{a}}_{3} { }}}^{2} } \\ \end{array} } \right]$$.

r both RN models, the genetic (co)variance matrix (${\mathbf{A}}$) across the EC was obtaining using:

3 $${\mathbf{A}} = { }\phi {\mathbf{K}}\phi^{\prime}$$

In RN-L, $\phi$ was a 15 × 2 matrix containing a vector of 1’s in the first column and a vector of the EC values for each environment. In RN-NL, ${\varvec{\upphi}}$ was a had an additional two columns (15 × 4) consisting of the EC values squared and cubed, respectively.

### RN overall breeding value and stability estimates

In both RN-L and RN-NL, the GEBV for the intercept (${{\varvec{a}}}_{0}$) was treated as the overall GEBV for each genotype. The slope GEBV (${\mathbf{a}}_{1}$) in RN-L captured how much the overall GEBV of each genotype changed across the EC and was therefore used as a GEBV for stability. This is the standard model for deriving stability from linear RN models (e.g. de Souza et al. 2020; Kusmec et al. 2017; Lacaze et al. 2009), so it was treated as the control measure of stability. None of the GEBVs in RN-NL could be directly interpreted as stability. Stability could only be estimated in RN-NL using the second scale-correction method (outlined later).

The slope GEBV (${\mathbf{a}}_{1}$) could include variation due to both scale-type and rank-type G × E. The following two methods aim to remove the variation due to scale-type G × E, so that the resulting GEBVs describe variation due to rank-type G × E across the EC.

The first method was derived from Falconer (1990). Here, the correlation between the intercept and slope is attributed entirely to scale-type G × E, whilst the remaining variation that is independent of the correlation is attributed to rank-type G × E. We can obtain GEBVs for the slope that are independent of the genetic correlation between the intercept and slope by using a genetic regression:

4 $${\mathbf{a}}_{1}^{*} = {\mathbf{a}}_{1} - \frac{{{ }\sigma_{{{\text{a}}_{1} {\text{a}}_{0} }} }}{{\sigma_{{{\text{a}}_{0} { }}}^{2} }}{\mathbf{a}}_{0}$$

where ${\mathbf{a}}_{0}$ and ${\mathbf{a}}_{1}$ are the GEBVs for the intercept and slope, respectively, ${\hspace{0.17em}\sigma \hspace{0.17em}}_{{\mathrm{a}}_{0} }^{2}$ is the variance in intercept, and ${\sigma }_{{{\mathrm{a}}_{1}\mathrm{a}}_{0}}$ is the covariance between the intercept and slope. The resulting GEBVs (${\mathbf{a}}_{1}^{\mathbf{*}}$) are the essentially the residuals of a genetic regression between the intercept and slope components. This method, referred to hereafter as the genetic regression, was only applied to RN-L, as it cannot be used for reaction norms with higher-order polynomials (i.e. RN-NL).

The second method also attempts to remove variation in the reaction norm slope arising from scale-type G × E whilst allowing the use of RN models with higher-order polynomials. Briefly, the GEBVs of genotypes along the EC are standardised to a constant genetic variance to remove scale-type G × E. After standardisation, the change in GEBV for each genotype across the EC is used to estimate stability. This method, hereafter referred to as the standardisation method, was applied independently to both RN-L and RN-NL.

In more detail, the GEBV for genotype *i* at EC value $\mathrm{t}$ in a RN model is a function of the GEBVs for each regression coefficient ($a_{0}$ … $a_{p}$) and was calculated as follows:

$${\text{GEBV}}_{it} = {\text{ a}}_{{0_{i} }} + {\text{ a}}_{{1_{i} }} \times {\text{t}} + \ldots + {\text{a}}_{{p_{i} }} \times {\text{t}}^{{\text{p}}}$$

Likewise, the genetic variance at the different values of ${\text{t}}$ can be calculated using ${\mathbf{A}} = { }\phi {\mathbf{K}}\phi^{\prime}$, as described in Eq. 3. The GEBV for each genotype at each value of ${\text{t}}$ can then be standardised (SGEBV) based on the genetic variance:

$${\text{SGEBV}}_{{i,{\text{t}}}} = { }\frac{{{\text{GEBV}}_{{i,{\text{t}}}} }}{{\sqrt {{\text{Va}}_{{\text{t}}} } }}$$

where ${\text{Va}}_{t}$ is the genetic variance at EC value $\mathrm{t}$. By performing this across the range of EC values in the data, the SGEBVs for each genotype can be plotted across the EC. The resulting standardised reaction norm describes the breeding value of each genotype across the EC whilst being adjusted for changes in the scale of the genetic variance. The SGEBVs only represent relative performance and do not carry units. Since the SGEBVs are not linear with respect to $\mathrm{t}$, there is not a simple measure for stability. A solution is to use the change in SGEBV over a specified interval across the EC to estimate the stability (${\mathbf{S}}$):

5 $${\text{S}}_{i} = \frac{{{\text{ SGEBV}}_{{i, {\text{t}}_{x} }} - {\text{ SGEBV}}_{{i, {\text{t}}_{y} }} }}{{{\text{t}}_{x} - {\text{t}}_{y} }}$$

where ${\text{t}}_{x}$ and ${\text{t}}_{y}$ represent the maximum and minimum EC values in the specified interval. It is therefore possible to estimate the stability of each genotype for different segments of the EC. This could be used to explore whether different genotypes are more sensitive to different parts of the EC. The current study used an interval containing the entire range of the EC. Therefore, $\mathbf{S}$ was interpreted for each genotype as the change in GEBV relative to the population across the range of the EC and was calculated using both RN-L and RN-NL.

### Factor-analytic models

Unlike RN models, FA models ‘find’ environmental variables, called common factors, that explain patterns of G × E across environments. The regression on these common factors can be used to estimate the stability of genotypes across environments. If the EC used in the RN models is a major driver of G × E, we can expect estimates of stability and overall performance to be similar between these methods. Since FA models have special properties which allow the user to measure the amount of scale-type G × E in the regression coefficients for the environmental variables, we used stability estimated from FA models as a reference to compare the effectiveness of the two RN correction methods to account for scale-type G × E.

The first FA model (FA-2) used two common factors to model genetic effects that were common across the environments, along with specific genetic and residual variances for each of the 15 environments. FA-2 was described in matrix notation as follows:

6 $${\mathbf{y}} = {\mathbf{Xb}} + {\mathbf{Z}}_{{\mathbf{g}}} \left( {{{\varvec{\Lambda}}}_{{\mathbf{a}}} \otimes {\mathbf{I}}_{{\mathbf{n}}} } \right){\mathbf{f}}_{{\mathbf{a}}} + {\mathbf{Z}}_{{\mathbf{g}}} {{\varvec{\updelta}}}_{{\mathbf{a}}} + {\mathbf{e}}$$

where ${\mathbf{y}}$, ${\varvec{X}}$ and ${\varvec{b}}$ were the same as in Eq. 1, ${\mathbf{Z}}_{\mathbf{g}}$ was a design matrix linking records to the random effects, ${{\varvec{\Lambda}}}_{\mathbf{a}}=[ {{\varvec{\uplambda}}}_{{\mathbf{a}}_{1}},{{\varvec{\uplambda}}}_{{\mathbf{a}}_{2}}]$ was a *b* × *2* matrix of estimated loadings for the *b* environments and first (${{\varvec{\uplambda}}}_{{\mathbf{a}}_{1}}$) and second (${{\varvec{\uplambda}}}_{{\mathbf{a}}_{2}}$) common factors, and ${\mathbf{I}}_{\mathbf{n}}$ was an *n* × *n* identity matrix, where *n* was the number of genotypes, ${\mathbf{f}}_{\mathbf{a}}= {({\mathbf{f}}_{{\mathbf{a}}_{1}}^{\mathbf{^{\prime}}},{\mathbf{f}}_{{\mathbf{a}}_{2}}^{\mathbf{^{\prime}}})}^{\mathrm{^{\prime}}}$ was a *n* × 2 vector of genotype regression coefficients for the first (${\mathbf{f}}_{{\mathbf{a}}_{1}}$) and second (${\mathbf{f}}_{{\mathbf{a}}_{2}}$) common factor, ${{\varvec{\updelta}}}_{\mathbf{a}}={({{{\varvec{\updelta}}}_{\mathbf{a}}^{\mathbf{^{\prime}}}}_{1,},{{{\varvec{\updelta}}}_{\mathbf{a}}^{\mathbf{^{\prime}}}}_{2},\dots , {{{\varvec{\updelta}}}_{\mathbf{a}}^{\mathbf{^{\prime}}}}_{\mathbf{b}})}^{\mathrm{^{\prime}}}$ was a *n* × *b* vector containing the specific genetic effects (${{{\varvec{\updelta}}}_{\mathbf{a}}}_{\mathbf{j}}$) for the *b* environments, and $\mathbf{e}$ was the residual variance. The residual variance was estimated independently for each environment.

The variance of the common factors and specific effects was modelled according to:

$$\left[\begin{array}{c}{\mathrm{f}}_{\mathrm{a}}\\ {\updelta }_{\mathrm{a}}\end{array}\right]\sim \mathrm{N}\left(\left[\begin{array}{c}0\\ 0\end{array}\right], \left[\begin{array}{cc}{\mathbf{I}}\otimes \mathbf{G}&\quad 0\\ 0&\quad {{\varvec{\uppsi}}}_{\mathbf{a}}\otimes \mathbf{G}\end{array}\right]\right)$$

where ${{\varvec{I}}}_{{{\varvec{c}}}_{{\varvec{a}}}}$ was an identity matrix of order two, $\mathbf{G}$ was the genomic relationship matrix as calculated for the RN models, and ${{\varvec{\psi}}}_{{\varvec{a}}}$ was a *n* × *n* diagonal matrix with elements ${\psi }_{aj}$, which were the specific genetic variances for individual environments.

The matrix $\mathbf{A}$, which describes the additive genetic variance and covariance between the *b* environments, was calculated following:

$$\mathbf{A}={{\varvec{\Lambda}}}_{\mathbf{a}}{{\varvec{\Lambda}}}_{\mathbf{a}}^{\mathbf{^{\prime}}}+{{\varvec{\uppsi}}}_{\mathbf{a}}$$

The second FA model (RR-2) was a reduced-rank implementation of FA-2. RR-2 was identical to FA-2, except that the specific genetic variances for each of the 15 environments (${\psi }_{aj}$) were assumed to be zero. RR-2 was fitted because RN models also implicitly assume that the specific genetic effects are zero. Therefore, the results from RR-2 should account for the effect of estimating specific genetic effects when comparing the RN and FA models.

FA models with more than two common factors were not considered. This was because RN models essentially capture the performance of a genotype in two components: intercept (overall breeding value) and slope (change in breeding value over a unit change in EC). Similarly, the first common factor in a FA model usually represents the overall breeding value (comparable to the intercept), with the second common factor capturing stability (comparable to the slope). Going beyond two common factors would make it difficult to compare stability between FA and RN models and was not required to achieve the objective of the study, which was to test how well the RN model scale corrections accounted for scale-type G × E. For reference, the two common factors accounted for on average 72.8% of the additive genetic variance (see results section), whilst fitting a third common factor explained an additional 9.14% (results not shown).

### Overall breeding value and stability in FA models

Breeding values for overall performance and stability for each genotype were derived from FA-2 and RR-2 based on the methods outlined in Smith and Cullis (2018). This involved first rotating the loadings and regression coefficients for each common factor using singular value decomposition to make them orthogonal. After rotation, the loadings were analysed to ascertain how much scale or rank-type G × E contributed to the regression coefficients for each common factor.

The sign of the loadings represents contrasts between the environments (Smith and Cullis 2018). Since all genotypes have the same intercept for each common factor (0,0), there is no re-ranking of genotypes (or no contrast) across a common factor when the loadings all have the same sign. In this case, the regression coefficients will exclusively capture scale-type G × E.

**Fig. 1:**
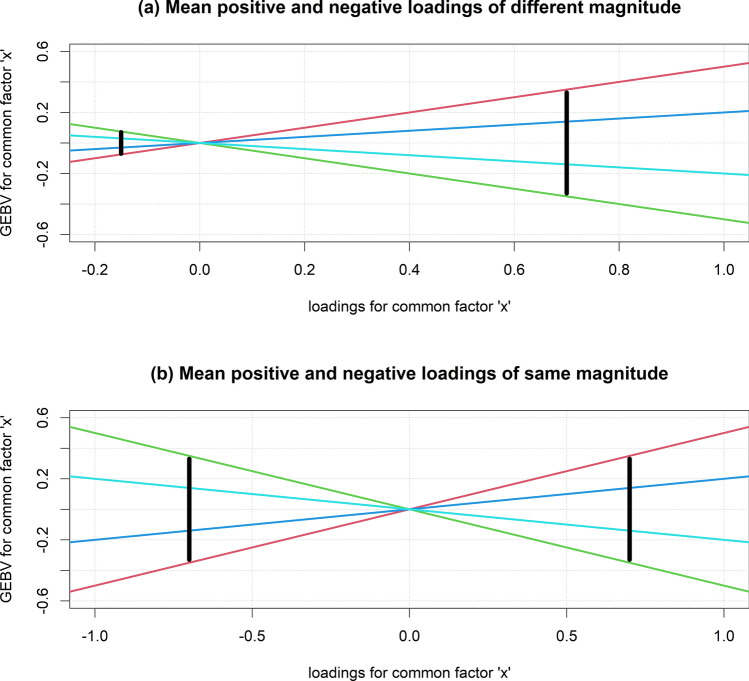
Schematic example of the regression of four genotypes (coloured lines) across a common factor, ‘x’ in a FA model. The vertical black lines represent the variation in GEBVs for the common factor ‘x’ at the average of the positive and negative loadings (or average contrasts). In **a**, the average negative and positive loading are − 0.15 and 0.7 units, respectively, and in **b**, − 0.7 and 0.7 units respectively

When there is a mix of positive and negative loadings, the regression coefficients capture at least some variation due to rank-type G × E, because the genotypes pass through (0,0) and re-ranking occurs. The degree to which the regression coefficients represent rank-type G × E can be assessed by comparing the absolute mean of the positive and negative loadings (or average contrasts), respectively, for the common factor. When the average contrasts are very different (Fig. 1a), most of the variation in the regression coefficients will be due to scale-type G × E, because the variance of GEBVs for the common factor will be very different between the average contrasts. When the absolute mean of the positive and negative loadings is equal, the variance of GEBVs for the common factor at the average contrasts will also be equal (Fig. 1b), so the variation in regression coefficients will be mostly due to rank-type G × E.

According to Smith and Cullis (2018), the loadings for the first common factor are usually the same sign, whilst the remaining common factors tend to be a mix of positive and negative loadings. This experience was reflected in our analysis (see results). This meant that the breeding value for overall performance across environments ($\mathbf{O}\mathbf{P}$) could be calculated as a function of the first common factor, since it represented a genetic effect common to all environments with no re-ranking. Therefore, $\mathbf{O}\mathbf{P}$ was calculated as the mean additive genetic effect across the loadings for the first common factor:

$${\mathrm{OP}}_{i}=\frac{1}{\mathrm{b}}\sum_{j=1}^{b}{\widetilde{\uplambda }}_{{\mathrm{a}}_{1j}}^{*}{\widetilde{\mathrm{f}}}_{{\mathrm{a}}_{1i}}^{*}$$

where ${\widetilde{\uplambda }}_{{\mathrm{a}}_{1j}}^{*}$ was the rotated loading for the first common factor for environment *j*, and ${\widetilde{\mathrm{f}}}_{{\mathrm{a}}_{1i}}^{*}$ was the rotated regression coefficient for genotype *i* on the first common factor.

Because the second common factors in FA-2 and RR-2 contained a mix of positive and negative loadings that were similar in magnitude, they captured variation in stability due to rank-type G × E. Therefore, the responsiveness of genotypes to the second common factor ($\mathbf{R}2$) was used as the GEBV for stability and was calculated as follows:

$${\mathrm{R}2}_{i}=({\overline{\uplambda } }_{2+}- {\overline{\uplambda } }_{\mathrm{a}2-}){\widetilde{\mathrm{f}}}_{{\mathrm{a}}_{2i}}^{*}$$

where ${\overline{\lambda }}_{2+}$ and ${\overline{\lambda }}_{2-}$ represent the mean of the rotated positive and negative loadings for the second common factor, respectively. A smaller value of $\mathbf{R}2$ indicated a genotype with greater stability in rank. The GEBVs for $\mathbf{R}2$ were used as the reference estimate of stability to which the various GEBVs for stability estimated from the RN models were compared.

The percentage of genetic variance in FA-2 that was explained by common factor *d* (either 1 or 2) in environment *j* in was calculated using:

$${\mathrm{v}}_{{\mathrm{a}}_{dj}}={100({\widetilde{\uplambda }}_{{\mathrm{a}}_{dj}}^{*})}^{2}/ \left(\sum_{s=1}^{2}{{(\widetilde{\uplambda }}_{{\mathrm{a}}_{sj}}^{*})}^{2}+ {\uppsi }_{{\mathrm{a}}_{j}}\right)$$

where ${\widetilde{\uplambda }}_{{\mathrm{a}}_{dj}}^{*}$ was the rotated loading for common factor *d* in environment *j*, and ${\uppsi }_{{\mathrm{a}}_{j}}$ was the specific genetic effect in environment *j*. The average value of ${\mathrm{v}}_{{\mathrm{a}}_{d}}$ across the *j* environments was used to calculate the mean genetic variance explained by the common factors.

## Results

### Model summary

The FA models provided a better fit to the data than the RN models based on the Akaike information criterion (AIC), Bayesian information criterion (BIC) and log likelihood (Table 1). The linear RN model (RN-L) was a significantly poorer fit that the cubic RN model (RN-NL) (ΔAIC = 139), whilst the reduced-rank factor-analytic model (RR-2) provided a poorer fit than the factor-analytic model (FA-2) (ΔAIC = 1037).

**Table 1:** Summary of model fit. The number of parameters excludes those which fixed to zero during the model fitting process

^a^Model	Number of parameters	^b^AIC	^c^BIC	Log likelihood
RN-L	18	− 8076.89	− 7947.08	4056.445
RN-NL	25	− 8215.64	− 8035.35	4132.818
RR-2	44	− 8565.06	− 8247.76	4326.532
FA-2	58	− 9601.79	− 9183.52	4858.896

^a^*RN-L* linear reaction norm; *RN-NL* Nonlinear reaction norm; *RR-2* Reduced-rank factor-analytic model with two common factors and zero specific genetic effects; *FA-2* Factor-analytic model with two common factors and specific genetic effects

^b^Akaike information criterion

^c^Bayesian information criterion

Overall, the first and second common factors in FA-2 explained 50.6 and 22.4% of the genetic variance across the environments, respectively (72.8% in total, Table S1). The percent of genetic variance explained by each common factor within the environments is also reported in Table S1. The genetic (co)variance of the random regression coefficients for RN-L and RN-NL are given in Table S2, along with correlations. The genetic correlation between the intercept and slope (*r*) in RN-L was high (0.76), indicating that genotypes with larger intercepts also tended to have larger slopes. Based on the *r*^*2*^, this also implied that 57.8% of the variation in slope was due to scale-type G × E, whilst 42.2% was due to rank-type G × E.

Despite the differences in model fit, the correlations between GEBVs for overall performance in the RN models (${\mathbf{a}}_{0}$) and FA models ($\mathbf{O}\mathbf{P}$) were high, ranging between 0.93 and 0.95 (Table 2). The lowest correlation between GEBVs for overall performance was between the RN models (0.90). Although not shown, the correlation between GEBVs for stability in FA-2 and RR-2 (**R2**) was also very high (0.96).

**Table 2:** Pearson correlations between GEBVs for overall performance in the RN models (${\mathbf{a}}_{0})$ and FA models ($\mathbf{O}\mathbf{P}$)

^a^Model	RN-L	RN-NL	FA-2	RR-2
RN-L	1	0.90	0.93	0.95
RN-NL		1	0.94	0.95
FA-2			1	0.99

^a^*RN-L* Linear reaction norm; *RN-NL* Nonlinear reaction norm; *FA-2* Factor-analytic model with two common factors and specific genetic effects; *RR-2* Reduced-rank factor-analytic model with two common factors and zero specific genetic effects

### Magnitude of G × E

**Fig. 2:**
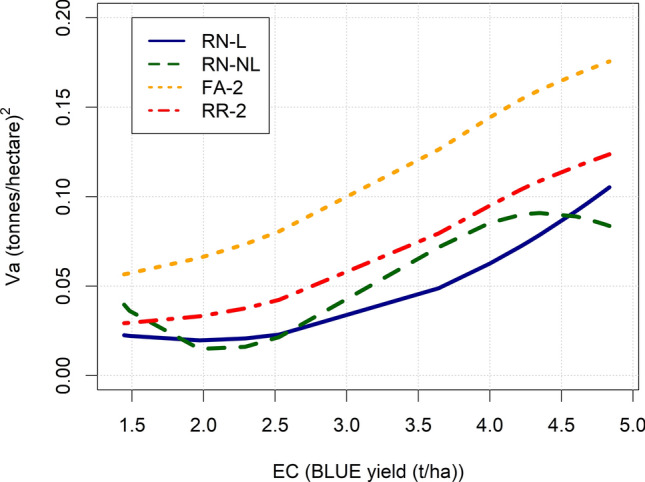
Additive genetic variance (Va) across the environmental covariable (EC) for the four models. In the RN-L and RN-NL, the line connects the estimates of Va for each environment, whilst a smoothing spline was fitted to the estimates of Va across the EC in RR-2 and FA-2. RN-L: linear reaction norm; RN-NL: nonlinear reaction norm; RR-2: reduced-rank factor-analytic model with two common factors and zero specific genetic effects: FA-2: factor-analytic model with two common factors and specific genetic effects

Genetic variance increased along the EC in RN-L and RN-NL by 430% and 486%, respectively, indicating strong scale-type G × E (Fig. 2). A smoothing spline fitted to the genetic variance of FA-2 and RR-2 also revealed similar increases in genetic variance across the EC. Overall, FA-2 estimated approximately 30–45% more genetic variance across the EC compared to RR-2, which was due to modelling specific genetic effects for each environment. The RN models estimated slightly less genetic variance across the EC compared to RR-2.

Genetic correlations between low and high yielding environments in the RN models were close to zero, indicating substantial levels of rank-type G × E (Table 3). The FA models tended to estimate lower genetic correlations between the environments.

**Table 3:** Minimum (min), maximum (max) and mean pairwise correlations between the 15 environments for the four models. The mean correlation was calculated using Fisher’s Z-transformation

^a^Model	RN-L	RN-NL	FA-2	RR-2
Min	0.08	0.02	– 0.43	– 0.51
Max	1.00	1.00	0.97	1.00
Mean	0.96	0.89	0.50	0.86

^a^*RN-L* linear reaction norm; *RN-NL* Nonlinear reaction norm; *FA-2* Factor-analytic model with two common factors and specific genetic effects; *RR-2* Reduced-rank factor-analytic model with two common factors and zero specific genetic effects

### Loadings of factor-analytic models

The average of the negative loadings for the first common factor was 92.0 and 90.4% smaller than the average of the positive loadings in FA-2 and RR-2, respectively (Table 4). Additionally, only one loading in RR-2 and two loadings in FA-2 were negative for the first common factor (Table S3). Therefore, the first common factor captured variation that was almost entirely due to scale-type G × E in both models. The average of the negative loadings for the second common factor were only 34.1 and 1.9% smaller than the average of the positive loadings in FA-2 and RR-2, respectively. Because the magnitude of the average positive and negative loadings was similar (especially in RR-2), the second common factor captured variation primarily due to rank-type G × E. Therefore, we can expect the regression on the second common factor, $\mathbf{R}2$, to capture variation due to rank-type G × E.

**Table 4:** Absolute value of the mean REML estimates of the positive and negative loadings for the two common factors in FA-2 and RR-2

^a^Model	Common factor	Mean positive loading	Mean negative loading
FA-2	1	0.273	0.022
	2	0.126	0.083
RR-2	1	0.231	0.022
	2	0.105	0.103

^a^*RN-L* Linear reaction norm; *RN-NL* Nonlinear reaction norm; *FA-2* Factor-analytic model with two common factors and specific genetic effects; *RR-2* Reduced-rank factor-analytic model with two common factors and zero specific genetic effects

### Comparison of stability measures

**Fig. 3:**
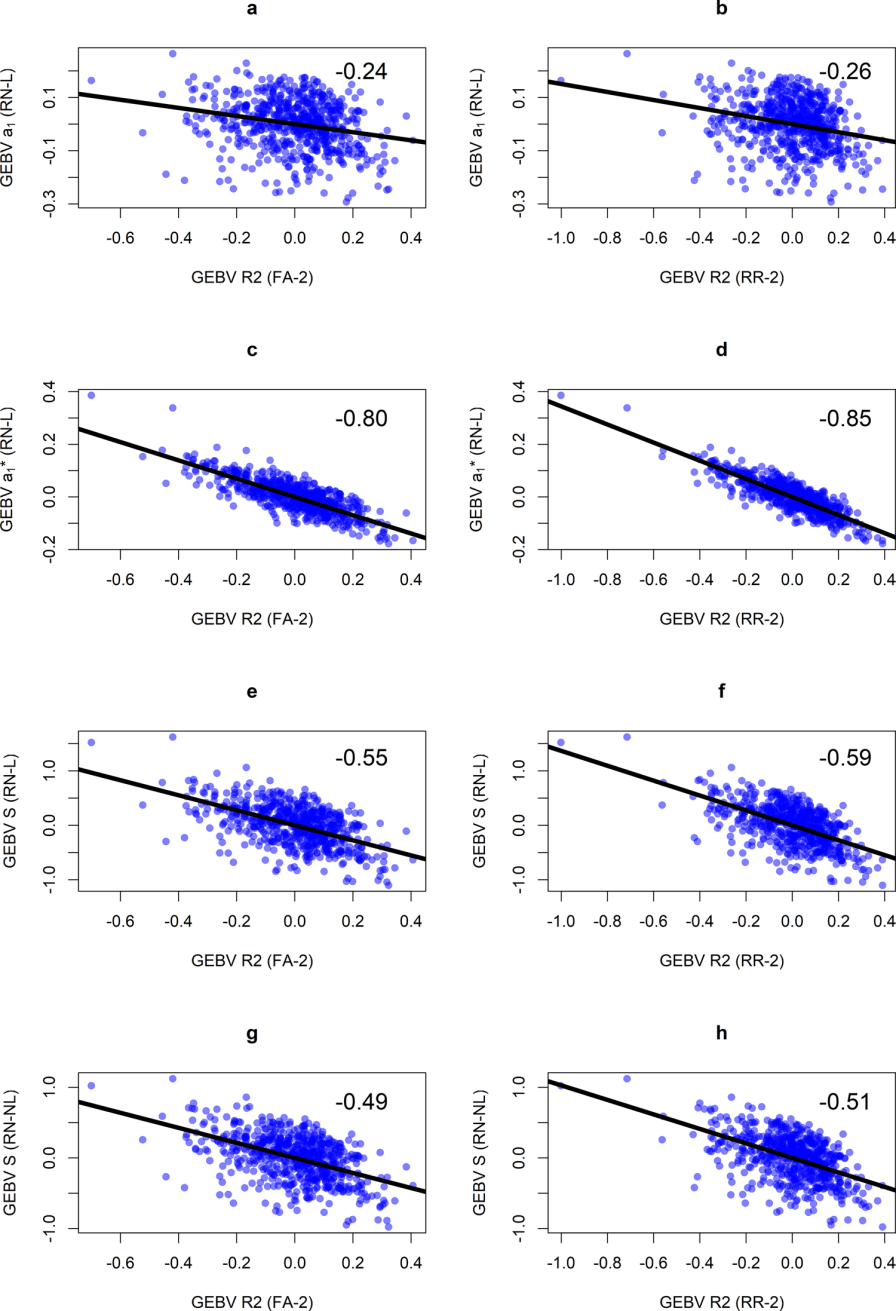
Scatterplots between stability GEBVs from RN models (${\mathbf{a}}_{1}$, ${\mathbf{a}}_{1}^{\mathbf{*}}$ and $\mathbf{S}$) compared with GEBVs for responsiveness to the second common factor (**R2**) in FA-2 and RR-2. The correlations are printed in each frame. RN-L: linear reaction norm; RN-NL: nonlinear reaction norm; FA-2: factor-analytic model with two common factors and specific genetic effects; RR-2: reduced-rank factor-analytic model with two common factors and zero specific genetic effects

The GEBVs for slope in RN-L (${\mathbf{a}}_{1}$) were lowly correlated with $\mathbf{R}2$ estimated in both FA-2 (−0.24) and RR-2 (−0.26) (Fig. 3a and b). Therefore, the standard method for determining stability in RN models gave very different results to the FA models. However, applying a genetic regression to the GEBVs (Eq. 4) for slope (${\mathbf{a}}_{1}^{\mathbf{*}}$) more than tripled the correlation with $\mathbf{R}2$, to −0.80 in FA-2 and −0.85 in RR-2 (Fig. 3c and d). Estimating stability after standardising the genetic variance in RN-L ($\mathbf{S}$) also increased the correlation with $\mathbf{R}2$ relative to ${\mathbf{a}}_{1}$, although the increase was more modest than ${\mathbf{a}}_{1}^{\mathbf{*}}$ (Fig. 3e and f). Using the same standardisation method with RN-NL ($\mathbf{S}$), which used cubic polynomials, did not increase the correlation with $\mathbf{R}2$ compared to RN-L (Fig. 3g and h). Note that the sign of the correlations is arbitrary, as the loadings and regressions in FA-2 and RR-2 can be multiplied by + 1 or − whilst retaining their meaning (Smith and Cullis 2018). They will be reported as absolute values herein.

### Relationship between overall performance and stability in different models

**Fig. 4:**
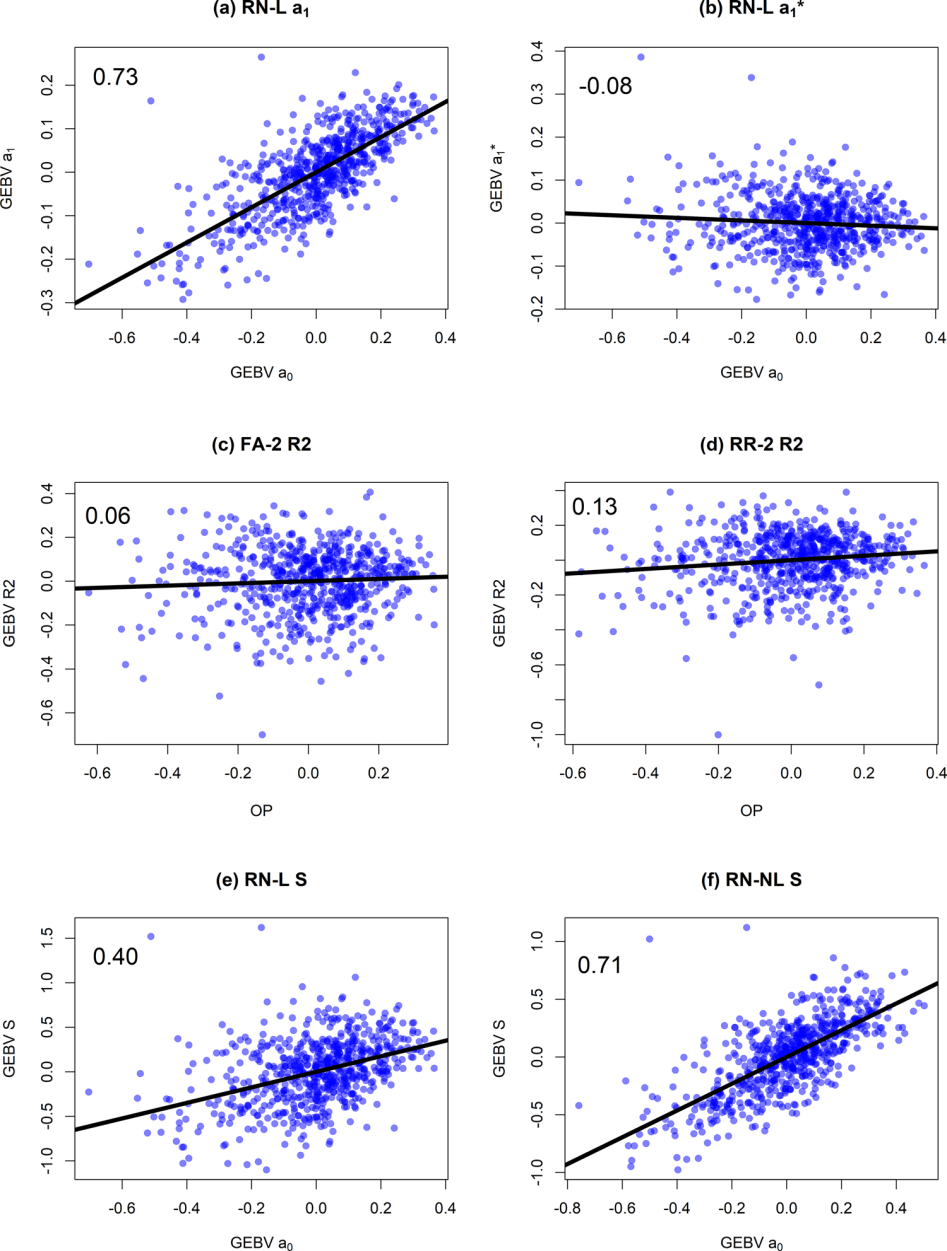
Relationships between overall performance (${\mathbf{a}}_{0}$ and $\mathbf{O}\mathbf{P}$) and stability (${\mathbf{a}}_{1}$, ${\mathbf{a}}_{1}^{\mathbf{*}}$, $\mathbf{S}$ and $\mathbf{R}2$) within the RN and FA models. The correlations are printed in each frame. RN-L: linear reaction norm; RN-NL: nonlinear reaction norm; FA-2: factor-analytic model with two common factors and specific genetic effects; RR-2: reduced-rank factor-analytic model with two common factors and zero specific genetic effects

In RN-L, the genetic correlation between the intercept and slope (0.76, Table S2) and the correlation between GEBVs for the intercept (${\mathbf{a}}_{0}$) and slope $({\mathbf{a}}_{1}$) (0.73, Fig. 4a) was high. This indicated that genotypes with a larger intercept tended to have larger change in GEBV across the EC and were therefore less stable. When the reaction norm slope was adjusted for scale-type G × E using the genetic regression (${\mathbf{a}}_{1}^{\mathbf{*}}$), the correlation with overall performance was close to zero (−0.08) (Fig. 4b). This was also the case in FA-2 and RR-2 (Fig. 4c and d). Although this was expected since ${\mathbf{a}}_{1}^{\mathbf{*}}$ and $\mathbf{R}2$ were both estimated orthogonally to the overall performance, the correlation between $\mathbf{S}$ and ${\mathbf{a}}_{0}$ was also much smaller than 0.73 and made no assumption about the relationship between the intercept and slope (RN-L: 0.40, Fig. 4e).

The correlation between $\mathbf{S}$ and ${\mathbf{a}}_{0}$ in the cubic RN-NL was comparably high (0.71, Fig. 4f). However, the individual standardised reaction norms in this model were very noisy (see next section). This could cause the estimates of $\mathbf{S}$ (and therefore the correlation with ${\mathbf{a}}_{0}$) to be very sensitive to small changes in the interval which it was evaluated over (${\mathrm{t}}_{x}$ and ${\mathrm{t}}_{y}$), making interpretation of the correlation difficult.

### Reaction norm scale-corrections methods

**Fig. 5:**
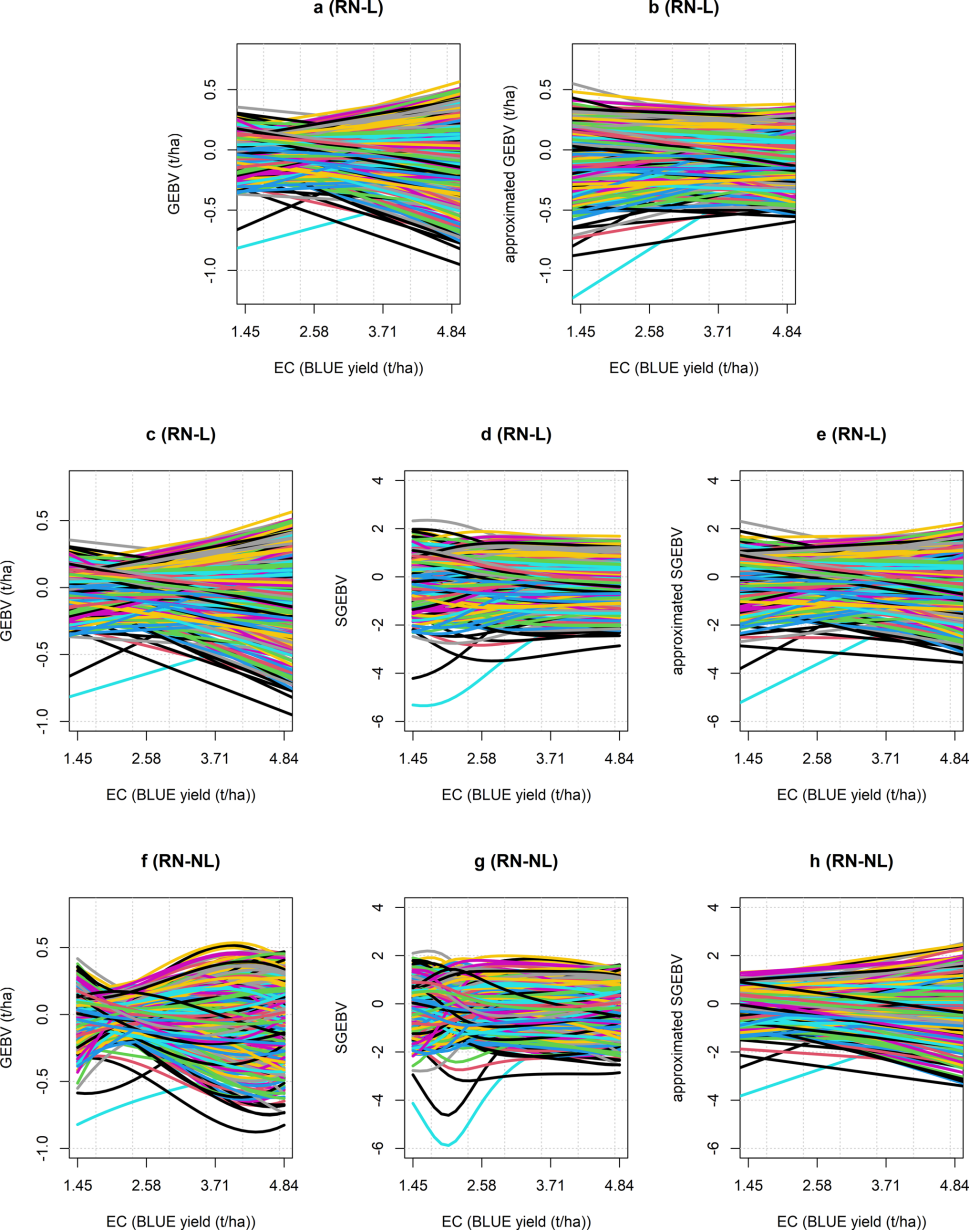
Reaction norms before and after applying the scale corrections. The top row shows the RN-L before **a** and after **b** applying the genetic regression, where the slope of each genotype is given by ${\mathbf{a}}_{1}$ and ${\mathbf{a}}_{1}^{\mathbf{*}}$, respectively. The middle row shows RN-L before **c** and after **d** standardisation, and a linear approximation of the standardised reaction norm **e** where the slope of each genotype is given by their average change in SGEBV across the entire EC. The bottom row shows RN-NL before (**f**) and after (**g**) standardisation, and a linear approximation of the standardised reaction norm (**h**) where the slope of each genotype is given by their average change in SGEBV across the entire EC. RN-L: linear reaction norm; RN-NL: nonlinear reaction norm

To explore reaction norm scale-corrections methods in more detail, the reaction norms for RN-L and RN-NL were plotted before and after applying the two correction methods (Fig. 5). The two genotypes with very poor performance at low EC levels (coloured blue and black, Fig. 5a, c) were ‘check’ varieties with lots of yield records and were not considered outliers in the analysis. Before the applying the correction methods, there was greater variation in GEBVs at higher mean yields and individual reaction norms crossed over, demonstrating both scale-type and rank-type G × E, respectively (Fig. 5a, c and f). When the slope of reaction norm was given by the genetic regression (${\mathbf{a}}_{1}^{\mathbf{*}}$), the variation in GEBVs was more constant across the EC, although there was slightly more variation in the low-yielding environments (Fig. 5b). This was likely because the mean EC (3.56 t/ha), which was the intercept, was slightly larger than the midpoint of the EC range (3.14 t/ha). The slope of regression changed direction for some genotypes.

When the GEBVs were standardised by the genetic variance, the reaction norms for RN-L were no longer linear, whilst the variation of GEBVs was also constant across the EC in both models (Fig. 5d and g). This can be seen more clearly in Fig. S2, which shows the variance of the GEBVs across the EC before and after standardisation. A linear approximation of the standardised reaction norms, which used the SGEBV when EC = 0 as the intercept and $\mathbf{S}$ as the slope, revealed that scale-type G × E was greatly reduced for RN-L (Fig. 5e) but to a lesser extent for RN-NL (Fig. 5h). The individual standardised reaction norms in RN-NL tended to be more erratic with very steep curves. This could indicate that the model was attempting to capture more complex G × E effects, rather than the general pattern across environments.

## Discussion

The MET dataset contained large differences in the genetic variance (scale-type G × E) and low genetic correlations (rank-type G × E) between environments across the EC, which is expected in populations where G × E is significant. This level and type of G × E is typical of METs collected in Australian plant breeding programs (Smith et al. 2021). Hence, the data were a good resource for testing the scale-correction methods in this study.

The standard estimate of stability in a RN model is the breeding value for the slope. The slope GEBVs $\left({\mathbf{a}}_{1}\right)$ were very lowly correlated (0.24 to 0.26) with stability GEBVs estimated in the FA models ($\mathbf{R}2$) (Fig. 3). This is interesting, as the RN and FA models would produce vastly different rankings of genotypes based on stability. Likewise, the SNP effects that underly the GEBVs would also be very different, which could lead to conflicting accounts of the genetic architecture of stability. However, correcting the slope GEBVs using a genetic regression $({\mathbf{a}}_{1}^{\mathbf{*}}$) tripled the correlation with $\mathbf{R}2$ (0.80 to 0.85). Because $\mathbf{R}2$ captured mainly rank-type G × E (Table 4), we can conclude that 1) ${\mathbf{a}}_{1}$ was lowly correlated with $\mathbf{R}2$ because it contained variation due to scale-type G × E and 2) ${\mathbf{a}}_{1}^{\mathbf{*}}$ was highly correlated with $\mathbf{R}2$ because the variation due scale-type G × E in ${\mathbf{a}}_{1}$ was successfully removed by the genetic regression.

The GEBVs estimated using the standardisation method ($\mathbf{S}$) were also more highly correlated with $\mathbf{R}2$ compared to ${\mathbf{a}}_{1}$, although the increase was less than with ${\mathbf{a}}_{1}^{\mathbf{*}}$. Therefore, it appears that the genetic regression is a more suitable approach for accounting for scale-type G × E in linear reaction norms. The correlation with $\mathbf{R}2$ was higher when $\mathbf{S}$ was estimated using the linear RN model (RN-NL) compared to the cubic RN model (RN-L). However, we would expect the linear reaction norm to be more closely related to $\mathbf{R}2$ than a cubic reaction norm, because $\mathbf{R}2$ is also a linear regression. Therefore, it was difficult to evaluate the merit of estimating $\mathbf{S}$ in either RN-L or RN-NL based on these results. Nevertheless, the standardisation method should still be useful to account for scale-type G × E in situations which require curvilinear reaction norms.

The intercept and slope GEBVs were highly correlated in RN-L (Fig. 4a, Table S2), indicating that genotypes with a larger intercept tended change more in GEBV across the EC and were therefore less stable. However, the correlation was close to zero when scale-type G × E was accounted for using a genetic regression and factor-analytic models (Fig. 4b, c and d), indicating there was no relationship between overall performance and stability due to rank-type G × E. This is similar to other analyses of stability which account for scale-type G × E (Smith and Cullis 2018; Tolhurst et al. 2019). This highlights the importance of understanding how stability is defined when interpreting its relationship with overall performance in different studies.

The additional benefit gained by applying the scale corrections is that the remaining slope variation should represent rank-type G × E, which arises when the genetic correlation between environments is less than unity. This could be used to explore how the genetic architecture of a trait varies across an EC. This phenomenon was highlighted in a reaction norm study in Australian sheep (Waters et al. 2022), where a seemingly pleiotropic quantitative trait loci affected both the intercept and slope but disappeared when a genetic regression was used. Instead, a new region which contained a group of genes previously associated with environmental sensitivity in livestock was identified. It would be interesting to see if similar reports of pleiotropic genes affecting overall performance and stability in plants using reaction norms (e.g. Li et al. 2019) also change if additional analysis to account for scale-type G × E is used.

There are exciting prospects for the improvement of yield stability in breeding programs using the genomic models in this study. Genomic breeding values derived from a training population such as routine METs could be used in combination with a speed-breeding program to fast track the creation of stable genotypes (Watson et al. 2018). In MET data, stability GEBVs derived from factor-analytic models would be recommended over the reaction norm models due to their superior ability for capturing the multifactorial causes of G × E in these settings (Kelly et al. 2007). This was realised in our analysis based on the AIC, BIC and the log likelihood. Additionally, FA-2 estimated between 30 and 45% more genetic variance within environments compared to the remaining models, indicating that it could disentangle G × E more efficiently. Despite this, the GEBVs from both models were highly correlated for overall performance (0.93–0.95) and stability (0.80 to 0.85) after scale-type G x E was accounted for. This is important, considering that the linear reaction norm required approximately two minutes to converge, compared to approximately 1 h 10 min (RR-2) and 6 h 4 min (FA-2) for the factor-analytic models. As the number of genotypes and environments in routine analysis grow, the value of using genomic reaction norms for their speed also could increase. Examples of reaction norm models being used to efficiently fit dense genomic analyses with hundreds of thousands of genotypes can be found in livestock and human studies (Carvalheiro et al. 2019; Lee and van der Werf 2016; Ni et al. 2019).

It is increasingly common for METs to collect environmental information to understand the causal factors underlying G × E (Cooper et al. 2021). A common way to incorporate the large number of resulting ECs is through (co)variance structures which describe the relationships between the environments based on the ECs (Jarquín et al. 2014; Raffo et al. 2022). Because these models implicitly regress phenotypes on ECs, the have also been interpreted as reaction norm models. Whilst these models have demonstrated increased predictive ability over models which lack ECs (Cuevas et al. 2017; Lopez-Cruz et al. 2015), it is unclear whether they can reveal the stability of genotypes across the ECs due to the implicit nature of their fit. If methods to extract the regressions of genotypes across the ECs become available, the impact scale-type G × E should be explored when estimating stability.

Another promising example for modelling this type of data integrates measured ECs into factor-analytic models (Tolhurst et al. 2022). In this study, the ECs explained mostly rank-type G × E, whilst the unobserved common factors explained scale-type G × E. Therefore, genotypes could be ranked based on their stability to the important ECs in the model. These models also outperformed equivalent reaction norms, which fit implicit random regressions on the individual environmental factors. Despite this, reaction norms should be the most efficient method for analysing stability when the EC is known and controlled (e.g. Houshmandfar et al. 2019; Paschke et al. 2003; Sadras et al. 2012), or the main source of G × E for the trait (Li et al. 2018; Millet et al. 2019).

## Conclusion

This study investigated the ability of two methods to partition variation in the slope of a reaction norm model into the two types of G × E (rank-type and scale-type) which underly it. Stability estimated from factor-analytic models, which explicitly disentangle rank-type and scale-type G × E, was used for comparison. The two methods substantially increased the correlation with stability estimated from the factor-analytic models, indicating that they removed variation in the reaction norm slope that originated from scale-type G × E. The genetic regression method appeared to be the most effective, yielding estimates of stability that were highly correlated (0.80—0.85) to those estimated from the factor-analytic models. Although the standardisation method had a more modest increase, it could still be useful in situations where curvilinear RN models are required. Analyses which use reaction norms should consider implementing the scale corrections outlined in this study to gain additional understanding of the nature of G × E and stability in their population.

## Supplementary Information

Below is the link to the electronic supplementary material.

Supplementary file1 (PNG 134 kb)

Supplementary file2 (PNG 351 kb)

Supplementary file3 (DOCX 15 kb)

Supplementary file4 (DOCX 14 kb)

Supplementary file5 (DOCX 14 kb)

## Data Availability

The data that support the findings of this study are available from InterGrain Pty Ltd, but restrictions apply to the availability of these data, which were used under licence for the current study, and so are not publicly available. Data are however available from the authors upon reasonable request and with permission of InterGrain Pty Ltd.
