# Supplementary figures and images for: Partitioning the forms of genotype-by-environment interaction in the reaction norm analysis of stability
Source: Theor Appl Genet. 2023 Apr 7;136(5):99. doi: 10.1007/s00122-023-04319-9 (PMC10082108; doi:10.1007/s00122-023-04319-9)

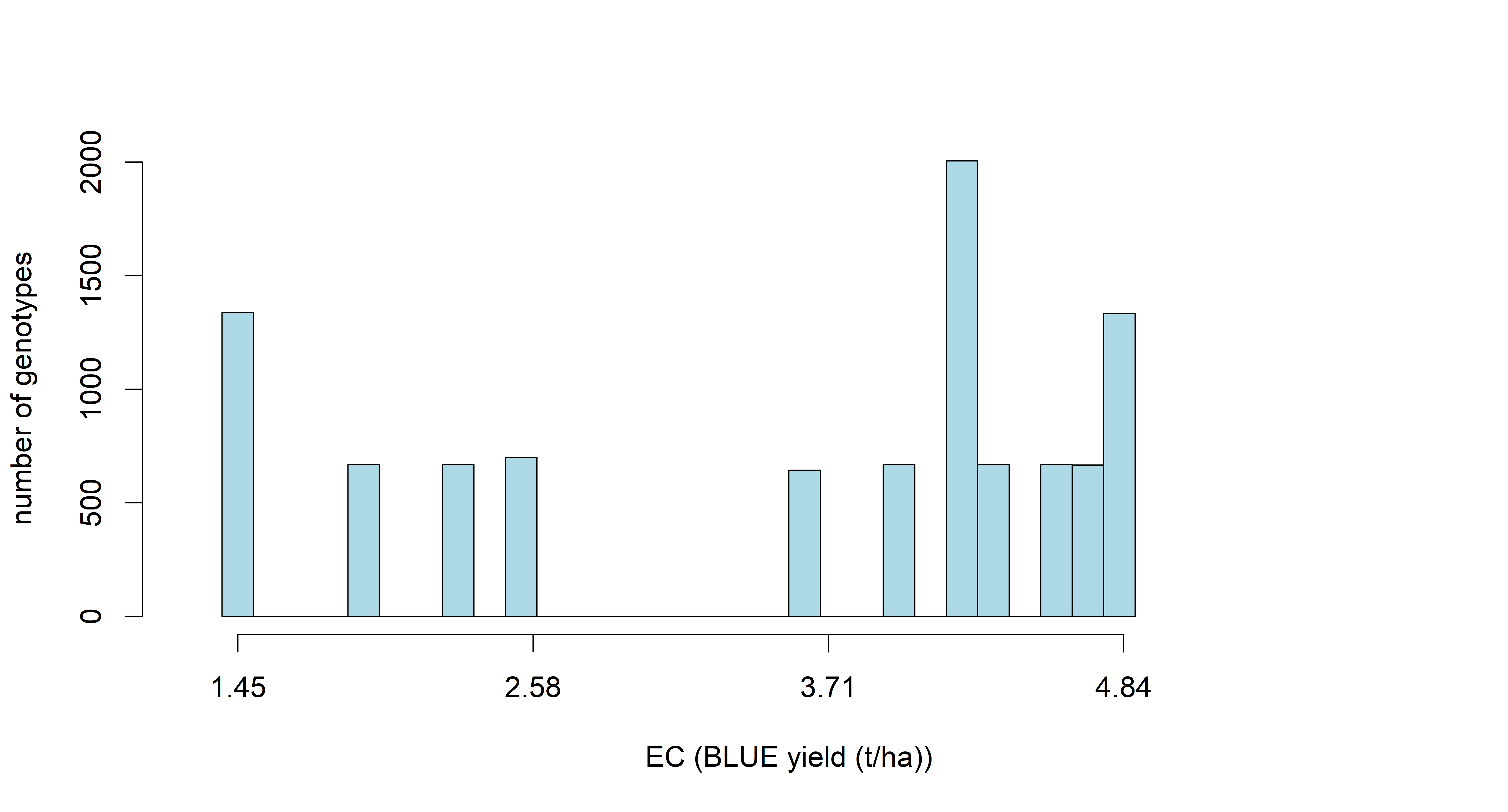

Supplement: Supplementary file 1 — Supplementary file1 (PNG 134 kb) [file 122_2023_4319_MOESM1_ESM.png]

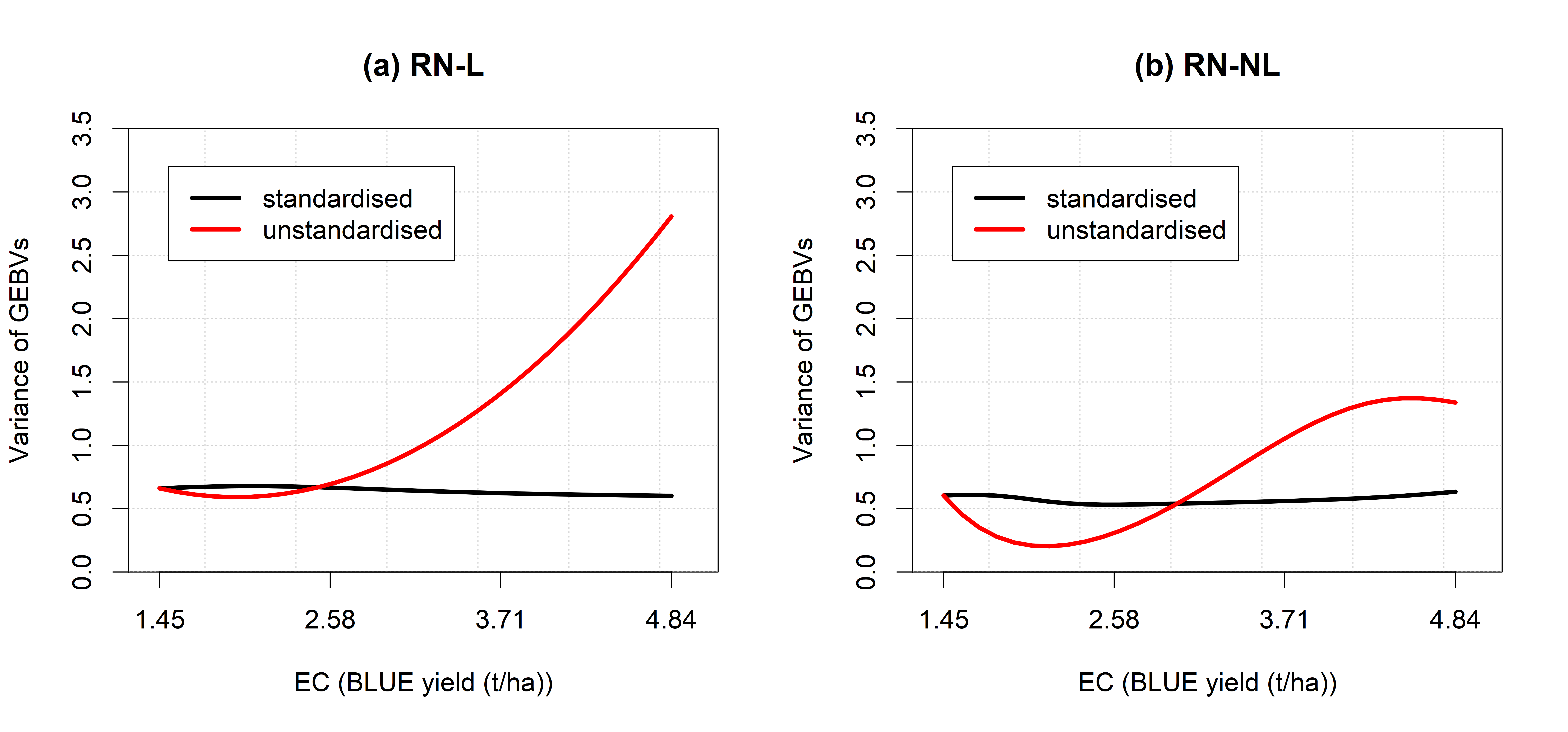

Supplement: Supplementary file 2 — Supplementary file2 (PNG 351 kb) [file 122_2023_4319_MOESM2_ESM.png]
