# Supplementary material for: Partitioning the forms of genotype-by-environment interaction in the reaction norm analysis of stability
Source: Theor Appl Genet. 2023 Apr 7;136(5):99. doi: 10.1007/s00122-023-04319-9 (PMC10082108; doi:10.1007/s00122-023-04319-9)
Supplement: Supplementary file 3 — Supplementary file3 (DOCX 15 kb) [file 122_2023_4319_MOESM3_ESM.docx]

| **Environment** | $\boldsymbol{v}_{\boldsymbol{a}_{\boldsymbol{1}}}$ | $\boldsymbol{v}_{\boldsymbol{a}_{\boldsymbol{2}}}$ | **Total (**$\boldsymbol{v}_{\boldsymbol{a}_{\boldsymbol{1}}}$ **+** $\boldsymbol{v}_{\boldsymbol{a}_{\boldsymbol{1}}}\boldsymbol{)}$ |
| --- | --- | --- | --- |
| 1 | 80.61 | 6.92 | 87.53 |
| 2 | 88.87 | 3.69 | 92.56 |
| 3 | 8.81 | 24.23 | 33.04 |
| 4 | 0.41 | 93.59 | 94.00 |
| 5 | 76.00 | 0.68 | 76.68 |
| 6 | 88.62 | 1.31 | 89.93 |
| 7 | 0.86 | 99.14 | 100 |
| 8 | 23.85 | 18.3 | 42.15 |
| 9 | 68.43 | 18.24 | 86.67 |
| 10 | 32.68 | 19.21 | 51.89 |
| 11 | 50.16 | 3.90 | 54.06 |
| 12 | 62.73 | 12.55 | 75.28 |
| 13 | 26.02 | 2.66 | 28.68 |
| 14 | 81.21 | 4.82 | 86.03 |
| 15 | 69.22 | 24.31 | 93.53 |
| **Average** | 50.56 | 22.24 | 72.80 |

**Table S1.** The percentage of genetic variance explained by the first
($v_{a_{1}}$) and second ($v_{a_{2}}$) common factor for each environment in FA-2.
