# Supplementary material for: Partitioning the forms of genotype-by-environment interaction in the reaction norm analysis of stability
Source: Theor Appl Genet. 2023 Apr 7;136(5):99. doi: 10.1007/s00122-023-04319-9 (PMC10082108; doi:10.1007/s00122-023-04319-9)
Supplement: Supplementary file 4 — Supplementary file4 (DOCX 14 kb) [file 122_2023_4319_MOESM4_ESM.docx]

**Table S2.** Variance (diagonal, bold), covariance (lower triangle) and correlations (upper triangle, italicised) for the random regression coefficients in RN-L and RN-NL.

| **Coefficients** | **RN-L** | | | |
| --- | --- | --- | --- | --- |
|  | $\mathbf{a}_{\mathbf{0}}$ | $\mathbf{a}_{\mathbf{1}}$ | $\mathbf{a}_{\boldsymbol{2}}$ | $\mathbf{a}_{\boldsymbol{3}}$ |
| $\mathbf{a}_{\mathbf{0}}$ | **0.0458** | *0.76* | *-* | *-* |
| $\mathbf{a}_{\mathbf{1}}$ | 0.0199 | **0.0151** | *-* | *-* |
| $\mathbf{a}_{\boldsymbol{2}}$ | - | - | **-** | *-* |
| $\mathbf{a}_{\boldsymbol{3}}$ | - | - | - | **-** |
| **Coefficients** | **RN-NL** | | | |
|  | $\mathbf{a}_{\mathbf{0}}$ | $\mathbf{a}_{\mathbf{1}}$ | $\mathbf{a}_{\boldsymbol{2}}$ | $\mathbf{a}_{\boldsymbol{3}}$ |
| $\mathbf{a}_{\mathbf{0}}$ | **0.0675** | *0.71* | *-0.75* | *-0.68* |
| $\mathbf{a}_{\mathbf{1}}$ | 0.0291 | **0.0260** | *-0.31* | *-0.87* |
| $\mathbf{a}_{\boldsymbol{2}}$ | -0.0212 | -0.0054 | **0.0119** | *0.46* |
| $\mathbf{a}_{\boldsymbol{3}}$ | -0.0091 | -0.0072 | 0.0026 | **0.0026** |
