# Supplementary material for: Partitioning the forms of genotype-by-environment interaction in the reaction norm analysis of stability
Source: Theor Appl Genet. 2023 Apr 7;136(5):99. doi: 10.1007/s00122-023-04319-9 (PMC10082108; doi:10.1007/s00122-023-04319-9)
Supplement: Supplementary file 5 — Supplementary file5 (DOCX 14 kb) [file 122_2023_4319_MOESM5_ESM.docx]

| **Environment** | **FAM-2** | | **RR-2** | |
| --- | --- | --- | --- | --- |
|  | ${\tilde{\boldsymbol{\lambda}}}_{\boldsymbol{a}_{\boldsymbol{1}}}^{\boldsymbol{*}}$ | ${\tilde{\boldsymbol{\lambda}}}_{\boldsymbol{a}_{\boldsymbol{2}}}^{\boldsymbol{*}}$ | ${\tilde{\boldsymbol{\lambda}}}_{\boldsymbol{a}_{\boldsymbol{1}}}^{\boldsymbol{*}}$ | ${\tilde{\boldsymbol{\lambda}}}_{\boldsymbol{a}_{\boldsymbol{2}}}^{\boldsymbol{*}}$ |
| 1 | 0.415 | **-0.122** | 0.336 | **-0.123** |
| 2 | 0.325 | 0.066 | 0.304 | 0.049 |
| 3 | 0.036 | 0.06 | 0.051 | 0.078 |
| 4 | **-0.013** | 0.192 | 0.001 | 0.176 |
| 5 | 0.48 | **-0.045** | 0.412 | **-0.117** |
| 6 | 0.513 | **-0.062** | 0.499 | **-0.071** |
| 7 | **-0.032** | 0.343 | **-0.022** | 0.27 |
| 8 | 0.098 | 0.086 | 0.048 | 0.055 |
| 9 | 0.316 | 0.163 | 0.333 | 0.107 |
| 10 | 0.073 | 0.056 | 0.08 | 0.023 |
| 11 | 0.296 | 0.083 | 0.329 | 0.113 |
| 12 | 0.291 | **-0.13** | 0.206 | **-0.099** |
| 13 | 0.175 | **-0.056** | 0.154 | 0.143 |
| 14 | 0.29 | 0.071 | 0.256 | 0.052 |
| 15 | 0.235 | 0.139 | 0.224 | 0.091 |

**Table S3.** Loadings of the common factors to each environment for FA-2 and RR-2. Negative loadings are highlighted in bold.
